# Spectrum of congenital anomalies of the kidney and urinary tract (CAKUT) including renal parenchymal malformations during fetal life and the implementation of prenatal exome sequencing (WES)

**DOI:** 10.1007/s00404-023-07165-8

**Published:** 2023-08-03

**Authors:** Josefine Theresia Koenigbauer, Laura Fangmann, Charlotte Reinhardt, Alexander Weichert, Wolfgang Henrich, Biskup Saskia, Heinz-Peter Gabriel

**Affiliations:** 1 Department of Obstetrics, Charité-Universitätsmedizin Berlin, Corporate Member of Freie Universität Berlin and Humboldt-Universität Zu Berlin, Berlin, Germany; 2 Prenatal Diagnosis Bergmannstrasse, Bergmannstrasse 102, Berlin, Germany; 3 Center for Human Genetics, Tuebingen, Germany

**Keywords:** CAKUT, PKD, DSD, Exome sequencing, WES, Detection rate, Polycystic kidney disease, Ectopic kidney, Horseshoe kidney, Urinary tract dilation

## Abstract

**Objectives and background:**

Congenital malformations of the kidney and urinary tract (CAKUT) have a prevalence of 4–60 in 10,000 livebirths and constitute for 40–50% of all end stage pediatric kidney disease. CAKUT can have a genetic background due to monogenetic inherited disease, such as PKD or ciliopathies. They can also be found in combination with extra-renal findings as part of a syndrome. Upon detection of genitourinary malformations during the fetal anomaly scan the question arises if further genetic testing is required. The purpose of this study was to determine the phenotypic presentation of CAKUT cases and the results of exome analysis (WES).

**Methods:**

This is a retrospective analysis of 63 fetal cases with a diagnosis of CAKUT or DSD at a single center between August 2018 and December 2022.

**Results:**

A total of 63 cases (5.6%) out of 1123 matched CAKUT phenotypes including renal parenchyma malformations. In 15 out of 63 WES analysis a pathogenic variant was detected (23.8%). In fetuses with isolated CAKUT the rate of detecting a pathogenic variant on exome sequencing was five out of 44 (11.4%). Ten out of 19 fetuses (52.6%) that displayed extra-renal findings in combination with CAKUT were diagnosed with a pathogenic variant.

**Conclusions:**

WES provides an increase in diagnosing pathogenic variants in cases of prenatally detected CAKUT. Especially in fetuses with extra-renal malformations, WES facilitates a gain in information on the fetal genotype to enhance prenatal counselling and management.

## What does this study add to the clinical work

**Table:** 

This study analyses cases of CAKUT and the rate of pathogenic variant in exome sequencing (WES). The results indicate that especially among fetuses with polycystic kidney disease or associated extra-renal malformations the rate of pathogenic finding is highest (29% and 52.6% respectively).	

## Introduction

Fetal abnormalities can be detected prenatally in around 2–3% of pregnancies [1, 2]. Upon detection, parental genetic counselling is offered together with invasive diagnostic procedures such as amniocentesis and chorionic villous sampling performing a karyotype and/or chromosomal microarray analysis (CMA). Overall, 8–10% of fetuses with an anomaly display an abnormal karyotype with an additional 6% of microdeletions and microduplications, which leaves most fetuses without a specific genetic diagnosis [3]. Depending on the publication, exome sequencing can detect a pathogenic variant in 20–80% of cases, if the karyotype or CMA is negative [4–7]. Prenatal WES can expand the yield of diagnosing an underlying disease in prenatally detected fetal abnormalities and it has the potential to increase the knowledge on prenatal disease [8].

Congenital anomalies of the kidney and urinary tract (CAKUT) including renal parenchymal malformations are detected in 20–30% of all fetal anomalies, identified during the prenatal scan [9]. The second trimester anomaly scan has become a standard of care provided for almost all risk pregnancies in Germany [10]. Some urinary tract malformations are detected during the third trimester scan, as a form of late onset CAKUT.

**Fig. 1:**
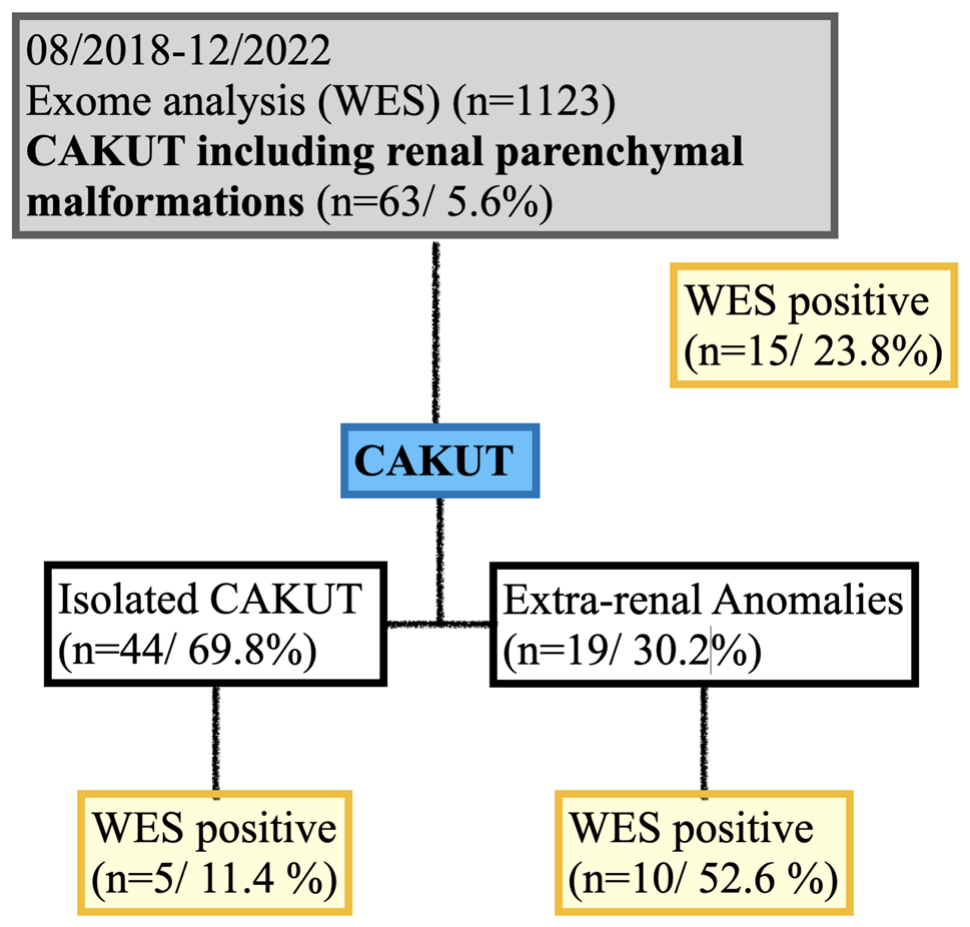
Description of the study population grouped according to the fetal phenotype. A total of 63 cases were analyzed during the investigation period (08/2018 – 12/2022)

CAKUT is a heterogeneous group of fetal malformations that affect the development of the kidneys and their outflow tracts (Fig. 1) [11, 12]. The prevalence is estimated to be around 4–60 in 10,000 births [13]. Extra-renal anomalies are detected in 30% of infants with CAKUT [9].

The development of the urinary tract is a multistage process, that is initiated by the ureteric bud and the metanephron at five gestational weeks. Any disturbance at any stage of the renal development can lead to different types of CAKUT with more severe defects occurring based on disruptions during early embryonic development [14–16]. The abnormal embryonic kidney development can be caused by renal parenchymal malformations (e.g., congenital cystic kidney disease), abnormal renal migration (e.g., altered position of the renal tissue) and disturbance in the developing renal collecting system (e.g., urinary tract obstruction) [17, 18]. CAKUT can be divided into non-genetic and genetic origins [19]. Certain teratogens, including drugs are known to cause an impairment of kidney development (e.g., ACE inhibitors and warfarin) [20]. The spectrum of CAKUT can range from almost no impairment to end stage renal disease requiring kidney transplantation or to a lethal condition due to pulmonary hypoplasia [15, 21]. Therefore, early detection during the prenatal ultrasound is essential to facilitate parental counselling and a close fetal and neonatal follow-up. Today, children affected by CAKUT have better chances of survival due to an improvement in early diagnosis, interventions and dialysis as well as kidney transplantation. Depending on the type of CAKUT severe comorbidities can be associated, impacting on overall survival and quality of life [19].

This study focuses on the additional information in diagnosing pathogenic variants, when performing WES after a negative karyotype and/or microarray upon detection of CAKUT prenatally.

### Materials and methodology

We searched the whole exome sequencing data bank at the Center of Human Genetics in Tübingen, Germany for cases of prenatally detected CAKUT including renal parenchymal malformations. A total number of 1123 WES were conducted from August 2018 until December 2022. Patient information was systematically reviewed including demographic factors, prior analysis and outcome. The genetic analysis was performed due to either CAKUT alone or combined with extra-renal findings concerning the fetal phenotype. As keywords we searched: CAKUT, polycystic kidney disease, hydronephrosis, megaureter, megacystis, LUTO, ureterocele, horseshoe kidney, ectopic kidney, fused kidney, kidney duplication, enlarged kidney, small kidney, hyperechogenic kidney. 63 cases out of 1123 (5.6%) matched the keywords. Descriptive analysis was performed applying SPSS version 22, IBM.

### Genetic analysis

Trio exome sequencing of our own cohort DNA quantity and quality were determined using Qubit Fluorometer and NanoDrop ND-8000 (Thermo Fisher Scientific, Dreieich, Germany). Enrichment of the coding DNA sections of the test persons was carried out with one of the following enrichment methods: SureSelect Human All Exon 50 Mb V6 Kit, SureSelect Human All Exon 50 Mb V7 Kit (Agilent, Santa Clara, CA, USA), the Twist Human Core + Refseq exome (Twist Bioscience, San Fransisco, CA, USA) or the CeGaT Exome Xtra V1 (CeGaT GmbH, Tübingen, Germany). For Agilent enrichment kits, sequencing libraries were prepared using the SureSelectXT workflow. Library preparation and capture for all samples was performed according to the respective manufacturer’s instructions. Paired-end sequencing was performed using the Novaseq6000 system (Illumina, San Diego, CA) with 2 × 100 base pairs (bp) read length. Sequence data were processed with Illumina bcl2fastq2. Adapter sequences were removed with Skewer and the sequences obtained were aligned to the human reference genome (hg19) with the Burrows Wheeler aligner (BWA mem). Sequences that could not be clearly assigned to a genomic position were removed, as were sequence duplicates that were probably due to amplification (internal software). The average coverage was 170x. Sequence variants (single nucleotide exchanges and short insertions/deletions) were determined from the remaining high-quality sequences (CeGaT StrataCall). Copy number variations (CNV) were computed on uniquely mapping, non-duplicate, high quality reads using an internally developed method based on sequencing coverage depth. Briefly, we used reference samples to create a model of the expected coverage that represents wet-lab biases as well as intersample variation. As a prediction software Mutationtaster, SpliceAI and NSFP was utilised parallely.

## Results

From August 2018 until December 2022 a total of 63 cases (5.6%) out of 1123 were analysed. The Center for Human Genetics received fetal DNA from all over Germany. The fetuses received a primary diagnostic ultrasound scan at a specialised prenatal diagnosis unit. The prenatal scan was conducted as a systemic evaluation of the fetal anomaly including echocardiography based on international recommendations [22–25]. The mean maternal age was 33.2 years (range 19–41 years, SD ± 5.14). The mean gestational week at testing was 23.2 (range 14–35, SD ± 5.32). In 11 studies (17.4%) exact data on GW was missing. 3.2% (2) of DNA samples were obtained postmortem. 7.9% (5) of fetuses were diagnosed during the first trimester, 52.4% (33) during the second trimester and 19.1% (12) in the third trimester. In 96.9% (61) the DNA sample was derived from a prenatal sample [amniocentesis in 58 cases (92.1%), chorionic villous sampling in three cases (4.8%)] and postmortem DNA sampling in two cases (3.1%). No confined placental mosaicisms (CPM) was detected among our cohort. The initial genetic test that was requested upon detection of a fetal malformation was a karyotype in 69.8% and a chromosomal microarray analysis in 28.6% (in one case the initial genetic test was not specified) (Table 1). In our cohort, the initial genetic test did not reveal a pathological finding, and therefore, further analysis was performed. Incidental findings were reported in five out of the 63 cases (7.9%). A variant of unknown/uncertain significance (VUS) was detected in two fetuses (3.2%). A trio exome sequencing (trio WES) was conducted in 61 cases (96.8%). In two fetuses a single WES was performed. The mean turnaround time was 13.3 days (median 12 days, range 6–42 days, SD ± 6.02). Sanger validation of variants was performed in 98.4%.

**Table 1:** Baseline data on genetic testing in numbers and percentage or mean and median

	*n*	Percentage	Mean; median (range)
Initial genetic testing			
Karyotype	44	69.8%	
Chromosomal microarray analysis	18	28.6%	
Not specified	1	1.6%	
Incidental findings			
None	58	92.1%	
Reported	5	7.9%	
Variant of unknown significance detected			
None	61	96.8%	
Reported	2	3.2%	
Type of exam			
Single	2	3.2%	
Duo	0	0.0%	
Trio	61	96.8%	
Type of NGS			
Clinical ES	0	0.0%	
WES	63	100.0%	
WGS	0	0.0%	
Turnaround time NGS (in days)			13.3; 12 (6–42)

*NGS* next generation sequencing, *WGS* whole genome sequencing

### Detection rate

In 15 out of 63 WES analysis a pathogenic variant was detected (23.8%) (Fig. 1, Table 2, figure). In fetuses with isolated CAKUT the rate of detecting a pathogenic variant by exome sequencing was five out of 44 (11.4%). Ten out of 19 fetuses (52.6%) that displayed extra-renal findings in combination with CAKUT were diagnosed with a pathogenic variant. The associated findings were of the fetal face (cleft palate), the central nervous system (vermian hypoplasia, dilated cisterna magna), of the neck (lateral cervical cysts), thorax (diaphragmatic hernia, pulmonary hypoplasia), heart (not specified), abdomen (omphalocele, gastroschisis, microcolon), extremities (club feet, arthrogryposis), fetal hydrops and genital malformation (hydrometrocolpos, cloacal malformation). In addition, there were associated abnormal findings of the fetal growth (IUGR, macrosomia) and the amniotic fluid (polyhydramnios, oligohydramnios, anhydramnios). Three pregnancies underwent termination, among these was a case of bilateral renal agenesis, and two fetuses with LUTO.

**Table 2:** Specific detection rate of pathogenic variants for each fetal phenotype ± associated malformations divided into two groups: isolated CAKUT and non-isolated CAKUT

CAKUT	Isolated CAKUT			Non-isolated CAKUT		
	Total	Proportion with abnormal genetic testing (%)	Pathogenic variant	Total	Proportion with abnormal genetic testing (%)	Pathogenic variant
Renal agenesis (*n* = 14)						
Renal agenesis bilateral (*n* = 7)	4	1 (25%)	*HNF1B, TBC1D1*	3	0	
Renal agenesis unilateral (*n* = 7)	6	0		1	1 (100%)	*Hypomethylation C2*
Renal hypoplasia (*n* = 5)	3	0		2	2 (100%)	*GREB1L*
						*POGZ*
PKD (*n* = 20)	14	4 (29%)	*PKHD1*	6	3 (50%)	*INVS*
			*PKHD1*			*TP63*
			*PKD1*			*CEP290*
			*ETFA*			
Renal hyperplasia (*n* = 3)	3	0		0	0	
Hyperechogenic kidneys (*n* = 1)	1	0		0	0	
Horseshoe kidneys (*n* = 7)	5	0		2	1 (50%)	*KMT2A*
Hydronephrosis (*n* = 7)	4	0		3	2 (67%)	*FREM1*
						*KANSL1*
LUTO (*n* = 6)	4	0		2	1 (50%)	*CHD7*
	44	5 (11.4%)		19	10 (52.6%)	

In fetuses with isolated renal anomalies, the highest detection rate of pathogenic variants was among fetuses with isolated polycystic kidney disease (29% PKHD1, PKHD1, PKD1, ETFA). One fetus out of four with bilateral renal agenesis was diagnosed with HNF1B (25%). All other isolated CAKUT cases did not reveal a pathogenic variant in WES (Table 2).

A higher detected rate was found in fetuses with CAKUT and extra-renal findings: 10 out of 19 fetuses (52.6%) were diagnosed with a pathogenic variant. Three of six fetuses with polycystic kidney disease and extra-renal abnormalities displayed a pathogenic variant (INVS, TP63, CEP290). Some of the CAKUT cases with associated malformations did reveal a pathogenic variant in WES (unilateral renal agenesis Hypomethylation C2; horseshoe kidney KMT2A; hydronephrosis FREM2, KANSL1; LUTO CHD7; renal hypoplasia GREBIL, POGZ).

### Specific genetic findings (additional material)

The definitive genetic variants detected in the affected fetuses are listed in Table 3. Polycystic kidney disease (PKD) is the most common inherited kidney disease [26, 27]. There are two types: the frequently encountered autosomal dominant polycystic kidney disease (ADPKD) and more rare autosomal recessive polycystic kidney disease (ARPKD) as the most common types of monogenic cystic kidney disease. ADPKD often becomes apparent as adults, whereas ARPKD appears at a very young age or even during pregnancy as it is accompanied by an early and severe impairment of kidney function. Both types can lead to chronic kidney disease (CKD) and end-stage renal disease (ESRD). ADPKD is the most frequent PKD with a prevalence of 1:400–1000 deliveries [27]. Extra-renal anomalies are described and manifest in the liver, the blood vessels and the heart. Mortality is most often caused by cardiac complications or infectious diseases during renal replacement therapy. ADPKD is associated with autosomal dominant PKD1 or PKD2 loss [27, 28]. PKD2 loss is described to have a milder phenotype than PKD1 loss associated with a lower incidence of ESRD [26]. Prenatally ADPKD is associated with enlarged bilateral kidneys with or with hyperechogenicity [29]. As a differential diagnosis ADPKD, congenital nephrosis of the finnish type, Meckel Gruber syndrome, Trisomy 13 and Beckwith Wiedemann, as well as loss of HNF1B can appear prenatally with a similar phenotype with enlarged hyperechogenic kidneys [30].

**Table 3:** Specific gene variants detected on WES, with inheritance and classification of the variant

Syndromes	Affected gene	Classification of the variant	Inheritance/de novo	Type of NGS	Initial genetic testing	CAKUT ultrasound finding	Other malformations
Polycystic kidney disease 1	PKD1: c.8333dupG; p.Glu2779Argfs*43	Significant	Paternal inheritance	WES (trio)	Karyotype	Bilateral polycystic kidneys	None
Polycystic kidney disease 4	PKHD1: c.1486C > T; p.Arg496*; c.8206T > G; p.Trp2736Gly	Significant	Paternal and maternal inheritance	WES (trio)	Karyotype	Bilateral polycystic kidneys	None
Polycystic kidney disease 4	PKHD1: c.11438delT; p.Phe3812Serfs*7	Significant	Maternal inheritance	WES (trio)	Karyotype	Bilateral polycystic kidneys	None
Glutaric Acidemia IIA	ETFA: c.15_25dup; p.Gln9Argfs*20	Significant	Paternal and maternal inheritance	WES (trio)	Karyotype	Bilateral polycystic kidneys	None
Spectrum of TP63-related disorders	TP63: c.1717_1719dupATC; p.Ile573dup	Significant	De Novo	WES (trio)	Karyotype	Megaureter hydronephrosis unilateral polycystic kidneys	Cleft palate
Infantile Nephronophtis	INVS: c.1417delG; p.Ala473Glnfs*37	Significant	Paternal And Maternal Inheritance	WES (trio)	Karyotype	Bilateral polycystic kidneys	Situs Inversus
Joubert Syndrome 5	CEP290: c.5813_5817delCTTTA; p.Thr1938Asnfs*16 hom	Significant	Paternal And Maternal Inheritance	WES (trio)	Karyotype	Bilateral polycystic kidneys	Vermian hypoplasia dilated cisterna magna
BNAR Syndrome	FREM1: c.2078 + 1G > T; p.?; c.3274 + 4A > G; p.?	Significant	Paternal And Maternal Inheritance	WES (trio)	Karyotype	Urogenital sinus hydronephrosis unilateral renal agenesis	Hydrometrocolpos cloacal malformation
Koolen-De Vries Syndrome	KANSL1: c.1534-1G > C; p.?	Significant	De Novo	WES (trio)	Karyotype	Hydronephrosis	Hydrops lateral cervical cysts
CHARGE Syndrome	CHD7: c.5405-7G > A; p.?	Significant	De Novo	WES (trio)	Karyotype	Megacystis	Multiple Malformations (Not Specified)
Renal Cysts and Diabetes Syndrome (HNF1B), congenital Anomaly of the Kidneys and the Genitourinary Tract (TBC1D1)	HNF1B; c.1006C > G; p.His336Asp; TBC1D1: c.1326G > C; p.Gln442His	Variant of unknown significance	Unknown	WES (single)	Karyotype	Bilateral Renal Agenesis	None
White-Sutton Syndrome	POGZ: c.2513_2514insT; p.Ser839Leufs*25	Significant	De Novo	WES (trio)	Karyotype	Bilateral kidney hypoplasia	Club feet
Renal hypoplasia 3	GREB1L: c.1735C > T; p.Arg579*	Significant	Maternal Inheritance	WES (trio)	Karyotype	Bilateral kidney aplasia	Cardiac anomaly (not specified)
Beckwith-Wiedemann Syndrome	Hypomethylation IC2	Significant	De Novo	WES (trio) and Multiplex ligation-dependent probe amplification (MLPA)	Karyotype	Unilateral kidney agenesis	Facial anomaly (not specified) omphalocele
Wiedemann-Steiner Syndrome	KMT2A. C.7874G > T; p.Arg2625Leu	Significant	De novo	WES (trio)	Karyotype	Horseshoe kidneys	Cardiac and pulmonary anomalies (not specified)

Prenatally diagnosed findings of CAKUT and extra-renal findings

In our cohort, one case of Glutaric acidemia type II was detected (EFTA variant). It is an autosomal recessive syndrome caused by defects in electron transport flavoprotein (ETF) or ETF–ubiquinone oxidoreductase (ETF–QO) causing hypo- or nonketotic hypoglycemia and metabolic acidosis. Glutaric acidemia type II can be subdivided into 3 subtypes with different ages of onset, whereas early onset (in neonatal period) comes with the highest mortality. Patients have kidney abnormalities, hypotonia, cardiomyopathies as well as liver abnormalities, weakness, fatigue and myalgia [31]. The prevalence is 1:250,000 [32].

One fetus from our cohort was diagnosed with TP63-related disorder. TP63-related disorders are a spectrum of six autosomal dominant syndromes including Ankyloblepharon–ectodermal defects–cleft lip/palate (AEC) syndrome; Acro-dermo-ungual–lacrimal–tooth (ADULT) syndrome; Ectrodactyly, ectodermal dysplasia, cleft lip/palate syndrome 3 (EEC3); Limb–mammary syndrome; Split-hand/foot malformation type 4 (SHFM4) and Isolated cleft lip/cleft palate (orofacial cleft 8) [33–35]. Depending on the disorder affected individuals can display different combinations of ectodermal dysplasia (hypohidrosis, nail dysplasia, sparse hair, tooth abnormalities), cleft lip/palate, split-hand/foot malformation, syndactyly, oligodactyly and other limb anomalies, lacrimal duct obstruction, hypo- or hyperpigmentation, hypoplastic breasts and/or nipples, hypospadias, abnormalities of kidneys and urinary tract. Furthermore, affected individuals can show attached eyelids, skin erosions and erythema, scarring of the scalp, alopecia, trismus, multiplenfreckles and specific facial characteristics (hypoplasia of the maxilla, wide nasal root, small alae nasi and a small red of the upper lip). Most patients suffer from impaired hearing (AEC syndrome). The prevalence of T63-related disorders is not yet fully investigated [35].

Nephronophthisis (NPH) describes a heterogenous group of autosomal recessive inherited kidney disease that is associated with multiple cysts causing fibrosis, inflammation and conclusively ESRD. NPH belongs to the group of ciliopathies, and it can be categorized infantile, juvenile and adolescent depending on the age of onset. Juvenile NPH has an incidence of 1:50,000 to 1:1,000 000 [36]. The loss of the NPHP1 gene and more than 20 other genes causes NPH (Wolf). Extra-renal anomalies associated with NPH can be cerebellar malformations, ocular anomalies, hepatic fibrosis and skeletal malformations depending on the underlying genetic cause. In our cohort, INVS loss was detected, which caused nephronophthisis. NPH-associated syndromes are Meckel–Gruber syndrome, Bardel–Biedl syndrome, Joubert syndrome as well as Jeune syndrome and other rarer conditions [37–40].

In our cohort, there was one case of Joubert syndrome 5 (variant of CEP290). Classic Joubert syndrome (JS) is an autosomal recessive disease describing a combination of molar tooth sign (MTS, abnormality of cerebellum and brain stem), hypotonia and retardation of development [40, 41]. Furthermore, affected individuals possibly suffer from respiratory issues, such as tachypnea or apnea, eye abnormalities, ataxia and mental retardation. The prevalence of the Joubert syndrome and related disorders is 1:80,000–100,000. The prognosis of the syndrome mainly depends on the degree of respiratory disorders but also on the graduation of functional loss of kidneys and liver especially after apneas usually decrease within the first year of life [42].

One fetus was diagnosed with BNAR syndrome through an FREM1 variant. BNAR syndrome describes a rare autosomal recessive disorder characterized by hypertelorism, a bifid or broad nasal tip, short and bulky oral frenula with or without the presence of anorectal defects (anal stenosis, ventrally shifted anus) and renal dysplasia/agenesis without intellectual disability. This bifid nose was not detected in the fetus prenatally. In addition, there can be airway abnormalities. The prevalence is not known yet [43, 44].

Koolen-de Vries syndrome was detected in one fetus (KANSL1 variant). It is an autosomal dominant disorder affecting female and male individuals in the same way and frequency. The affected individuals show a combination of growth restriction, developmental delay (psychomotor, language development), intellectual disability, muscular hypotonia, feeding difficulties, typical facial characteristics and epilepsy. Furthermore, congenital malformations are described affecting the heart and the urogenital system. Life expectancy is not yet clearly defined; however, affected individuals usually reach adulthood. The prevalence of Koolen-de Vries syndrome is not fully investigated yet [45–47].

One fetus with multiple anomalies was diagnosed with CHARGE syndrome prenatally (CHD7 variant). It is a multi-systemic disorder. CHARGE is an abbreviation, standing for: coloboma, heart defects, atresia choanae, growth retardation, genital abnormalities, and ear abnormalities including deafness. CHARGE syndrome is detected in 1/10,000 newborn children with a heterogenous phenotype associated with a potentially poor outcome [48, 49].

Renal cysts and diabetes syndrome (RCAD) is caused by HNF-1ß-mutations. Inheritance is autosomal dominant, and the prevalence of the syndrome is not known. Kidneys show diverse sizes, most patients show enlarged kidneys prenatally and as a child, whereas affected adults often show a scaling down of the kidneys. The syndrome often leads to renal replacement therapy including kidney transplantation [50]. The onset and diagnosis of diabetes varies between 10 and 61 years. Extra-renal manifestations can be genital tract malformations, hyperuricaemia, gout as well as pathological liver function [51–53].

White–Sutton syndrome is an autosomal dominant disease caused by a POGZ variant encompassing a combination of intellectual disability, developmental retardation, facial dysmorphism, hypotonia, autism spectrum disorder and behavioral abnormalities [54, 55]. The facial appearance of the affected individuals is characterized by micro-/brachycephaly, a prominent forehead, hypertelorism, prognathism, downward facing corners of the mouth, expanded nasal bridge with frontally facing nostrils, low-set ears and palate abnormalities [56]. Affected individuals have been reported to reach more than 30 years of age and to have children.

Renal hypoplasia can be subdivided into four subtypes: segmental hypoplasia, Oligomeganephronia, simple hypoplasia and cortical hypoplasia. The inheritance follows autosomal dominant patterns. All types are considered to have less renal lobes compared to normal kidneys, different phenotypes of the kidneys. All types of renal hypoplasia may remain unknown for a long time, developing increasing loss of renal function [57]. All types are considered to have less nephrons compared to a normal kidney. In the majority of cases a normal contralateral kidney can be seen. The healthy kidney might compensate for the loss of function and grow larger than usual. All types of renal hypoplasia may remain unknown for a long time, developing increasing loss of renal function [57].

Beckwith–Wiedemann syndrome is a genomic imprinting disorder causing overgrowth and a predisposition to embryonal tumors, mostly Wilms tumor and hepatoblastoma [58–60]. The clinical phenotype is variable, often showing macroglossia and possible abdominal wall defects and abnormalities of liver, spleen, pancreas, kidneys and adrenals. The prevalence is 1:26,000 deliveries [61].

Wiedemann–Steiner syndrome (WSS) is detected in less than 1 out of 1,000,000 individuals [62]. The inheritance is autosomal dominant, and the age of onset can be between the prenatal period and childhood. The syndrome mainly describes the combination of growth restriction, developmental and cognitive retardation combined with a characteristic facial appearance. Apart from that, patients can be affected by epilepsy, ophthalmologic anomalies, congenital heart defects, hypotonia, vertebral anomalies, renal and uterine anomalies, dysfunction of the immune system, brain malformations and dental abnormalities. Developmental and cognitive delay shows very variable graduations. Overall, the effect on daily life of WSS on affected individuals varies from hardly noticeable symptoms to severe disabilities.

## Discussion

This study focuses on the detection rate of pathogenic variants in WES in fetuses with CAKUT including renal parenchymal malformations. The rate of prenatal CAKUT was relatively low with 5.6% among all performed WES at the data bank at the Center of Human Genetics in Tübingen, Germany. The overall detection rate of a pathogenic variant in our cohort was 23.8%. The highest yield of detection was among fetuses with extra-renal findings with 52.6% vs. 11.4% in fetuses with isolated CAKUT. A variety of pathogenic variants were detected with differing neonatal prognosis in our cohort (Table 2).

**Fig. 2:**
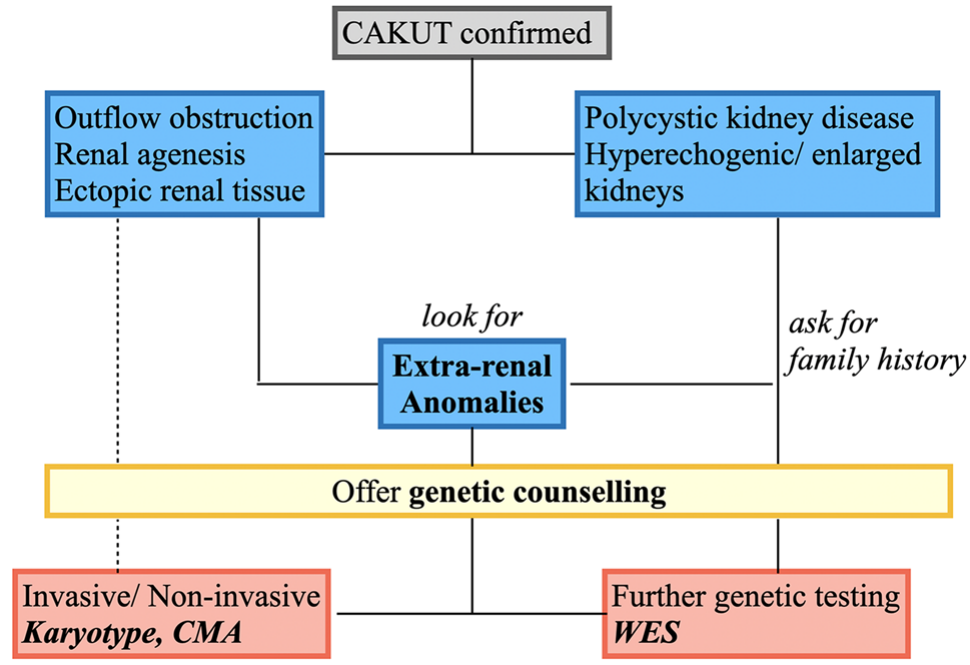
Counselling suggestion on further genetic testing upon prenatal detection of CAKUT: initially, it should be distinguished between structural/dysplastic kidneys (such as PKD, hyperechogenic kidneys) and outflow obstruction/renal agenesis and ectopic kidneys. Extra-renal abnormalities should be excluded. Genetic counselling should be offered. If simple outflow obstruction/renal agenesis and ectopic kidneys is apparent invasive/non-invasive genetic testing can be offered. In case of extra-renal anomalies invasive genetic testing is advisable. If karyotype/CMA is negative further testing as in WES should be discussed. If polycystic/hyperechogenic kidneys with or without extra-renal anomalies are detected prenatally invasive genetic testing should be discussed. Family history has to be taken into account. If the karyotype/CMA is negative further testing as in WES should be offered if available

In literature, WES can detect a pathogenic variant in 20–80% of cases when the karyotype or CMA is negative [4–7]. The diagnostic yield of WES in prenatal CAKUT has been described between 0% and 16% [63, 64]. Another publication supports our finding, that fetuses with extra renal anomalies display a higher diagnostic rate of pathogenic variants than fetuses with isolated renal findings (25% vs. 9.1%, respectively) [7]. We suggest the following diagnostic chronology (Fig. 2): initially, the sonographer should distinguish between structural/dysplastic kidneys (as in PKD, hyperechogenic kidneys) and outflow obstruction/renal agenesis and ectopic kidneys. Upon detection one should always search for extra-renal abnormalities. Genetic counselling should be offered. In case of simple outflow obstruction/renal agenesis and ectopic kidneys invasive/non-invasive genetic testing can be offered. In the case of extra-renal anomalies invasive genetic testing is advisable. If the karyotype/CMA is negative further testing as in WES should be discussed and offered. A recent study on 46 fetuses with megacystis and subsequent vesico-amniotic shunting (VAS), were able to distinguish between isolated (61%) and complex megacystis (39%) cases [65]. Interestingly, the rate of neonatal survival was much higher among the isolated mega cystic cohort (96.4% vs. 63.6% in the complex megacystis group). In our cohort, 6 fetuses with LUTO were identified, 2 displayed extra-renal findings out of which one fetus was diagnosed with CHD7 loss. In the future, further analysis should be conducted on genetic findings in fetuses with megacystis, to answer the question if complex megacystis case are associated with a complex genetic mutation. The answer will help facilitate counselling families prior to VAS.

If polycystic/hyperechogenic kidneys with or without extra-renal anomalies are detected prenatally invasive genetic testing is advisable. Family history should always be considered. If the karyotype/CMA is negative further testing-like WES should be offered.

Sequential sonographic follow-up and individualized care is required upon detection of CAKUT. Termination of pregnancy can be discussed and offered based on national legislation in cases with poor prognosis (e.g., lung hypoplasia due to renal mal-/dysfunction or specific pathogenic variants associated with unfavorable outcome).

This study has limitations such as missing data on the neonatal outcome and fallow-up. This issue should be addressed in future studies. Furthermore, some CAKUT cases are not highlighted in this study as they were not assessed via exam sequencing. Therefore, the resulting selection reduces the generalisability of this analysis. The rates of fetal anomalies may be overestimated. Further studies with larger cohorts are required to address this question.

## Conclusion

The wide availability of exome sequencing is revolutionizing our understanding of genetic causes of prenatal abnormalities including CAKUT. This analysis is so far the largest study on the implementation of WES in CAKUT. Upon prenatal detection of fetal CAKUT we suggest a thorough ultrasonographic scan to exclude associated extra-renal anomalies (Fig. 2). An individualized approach is necessary based on the sonographic findings (renal findings and extra-renal findings) considering the parental preference on finding out the underlying genotype. All patients should receive genetic counselling with the offer of genetic testing (non-invasive vs. invasive). If the karyotype or CMA is negative further genetic analysis can be offered (including WES). Based on our research, the highest detection yield of relevant pathogenic variants is among fetuses with renal and extra-renal findings (43.5% vs. 12.5% in fetuses with isolated CAKUT).

## Data Availability

The data supporting the findings of this study are available upon request.

## Abbreviations

CAKUT: Congenital malformations of the kidney and urinary tract
PKD: Polycystic kidney disease
DSD: Disorders of sex development
NIPT: Non-invasive prenatal testing
CAH: Congenital adrenal hyperplasia
WES: (Whole) exome sequencing
LUTO: Lower urinary tract obstruction
VUS: Variant of unknown/uncertain significance
DNA: Deoxyribonucleic acid
CMA: Chromosomal microarray analysis
MCKD: Multicystic kidney disease
